# Approved Societies and District Nurses

**Published:** 1913-05-17

**Authors:** 


### NATIONAL INSURANCE ACT DEPARTMENT.

### LIII.—Approved Societies and District Nurses.

We pointed out a fortnight ago that all classes of approved societies are beginning to suspect malingering on the part of a number of their members, and that they are considering the best means of preventing the misuse of sickness benefit. The worst offenders are apparently to be found among the members of women's societies. There are many reasons for this.

For instance, women's work in factories and workshops is, on the whole, much less regular than that of men, and the women are not, generally speaking, so entirely dependent for their support upon the wages they earn as are men. Furthermore, women's wages are on a lower standard than those of their husbands or brothers. Hence it follows that a payment of 7s. 6d. sickness benefit from her approved society is a far greater inducement to a woman to malinger than the larger payment of 10s. to a man. The malingering does not as a rule commence with an intention to defraud by the simulation of sickness. A woman is genuinely ill and incapacitated from following her usual employment. At the end of three days she is able to obtain the doctor's certificate that enables her to "declare on" the funds of her society, and in due course she receives her first week's benefit. This is sufficient to keep her from actual want for at least a few days, and while there is money in the house and domestic duties to perform she does not appreciate the necessity for returning to her usual employment in the factory or workroom. Having obtained the doctor's certificate and not having returned to work provides a seemingly valid excuse for continuing a claim to sickness benefit beyond its legitimate duration. Nothing could well be easier to carry into effect, and stringent measures are required to prevent unduly prolonged claims from becoming a permanent feature in the experience of women's societies.

### The Increase of Claims.

We had gathered certain information on the subject from one of the largest of the centralised societies, and the impression thereby gained that the experience was not confined to that class of approved society was confirmed by the announcement by one of the old friendly societies that its rate of claim for sickness benefit during the past three months had been 25 per cent. greater than in the corresponding quarter last year. Among a number of short paragraphs *apropos* to the subject in the current number of the monthly magazine of the Ancient Order of Shepherds we notice the following: "There has been more spring cleaning done in the daytime this year than has ever been known before in a time of good trade." It may be an advantage that the homes of the insured persons have had an extra thorough spring clean, but it is manifest that the funds of approved societies will not allow of an indefinite strain to make good the loss of wages thereby entailed!

The evil is recognised, but the difficulty is to prevent its occurrence. Sickness benefit is only payable, in accordance with the provisions of the Act, when the insured person is incapable of work, and household work is as much work as any other description, and if a woman who is ordinarily employed in a factory is attending to domestic duties, especially those of an arduous nature, instead of her normal occupation, she should not be allowed to draw on the sickness funds of her society.

### Female Sick Visitors.

Most of the old friendly societies appointed certain of their members as sick visitors and the practice to some extent continues, but the sick visitors are themselves at work during the day and the visits to members drawing benefit is extra evening work. For this reason the detection of a woman in the midst of her spring cleaning or of the washing, when she is supposed to be ill and incapable of work, by a sick visitor, who is precluded from calling until the evening, is a virtual impossibility. Again it has to be noted that in the provisions of the National Insurance Act relating to sick visitors it is definitely laid down that women shall not be visited otherwise than by women. This means that effective sick visiting can only be carried out in women's approved societies by the appointment of female sick visitors. But the insurance of women against sickness is still in its infancy, though brought into being on so large a scale, and it is no easy matter even for those old societies which have had sick visitors for years, while their members were men only, to find female members who are willing and competent to take up the duties of visitors in the interests of their societies.

In the case of the centralised societies—among which we may include the organisations formed in connection with the large collecting friendly societies and industrial assurance offices—there is even greater difficulty, for the representatives of these societies are men, and although they call to pay the sickness benefit they are debarred by the Act itself from acting in the capacity of visitors, so far as female members are concerned.

### Provisional Scheme for the County of Kent.

A method of combating the evil is about to be tried in Kent and it is understood that provisional arrangements have already been made, primarily with the object of preventing malingering, but also to some extent in order to provide better treatment for the insured persons when ill.

The idea, briefly, is for the approved societies—or as many of them as care to do so—to take advantage of Section 21 of the National Insurance Act, whereby they are empowered "to grant such subscriptions or donations as they may think fit . . . for the support of district nurses, and to appoint nurses for the purpose of visiting and nursing insured persons."

Nursing and the Approved Societies.

The initiative appears to have been taken by Queen Victoria's Jubilee Institute for Nurses. The Kent County Nursing Association is affiliated to the Institute, and it is through the former that the scheme is to be put into operation. The announcement by the Institute received an exceptionally favourable notice in the *Times* on May 9, though the point of view was rather different from that which we have just stated.

The advantages of efficient nursing are, of course, sufficiently recognised, but the approved societies cannot undertake expenditure on the provision of nursing for their members unless by so doing they are to receive a corresponding advantage in the reduction of claims for sickness benefit. To provide a nurse for each case of illness, or even for each member who is seriously ill, would be quite impossible for any approved society, and thus it is that the proposed arrangement is with district nurses, who will presumably be supplied with lists of the insured members who are ill and will be expected to call upon them, render such service as may be necessary, and report periodically to the society. Thus, there will result a more intimate acquaintance with the real nature of the illness upon which the claim for benefit is preferred than is at present possible with only the doctor's certificate, which enables the claim to be commenced, and the weekly reports from those who actually pay the money.

In course of time it may be possible for the movement to be extended in its operation so that really efficient nursing may be supplied, as well as medical attendance and treatment, to insured persons when they are ill. That time has not, however, yet arrived, and it must necessarily be deferred until larger funds are available for the purpose.

The Effect on Malingering.

In the meantime we welcome the proposals now seriously under discussion as a step in the right direction. That it is but a small step is apparent, for the sum suggested as the price which the societies are prepared to pay is no more than 3d. per member per annum. This is, of course, in the nature of an insurance premium, the payment being made for each member, whether ill or well, whereas only those members who are actually ill will receive the services of the nurses. If, however, as is expected and hoped, the visitation of members on the funds results in a lower rate of sickness claims the society as a whole will benefit and thus the payment for the district nurses will be justified.

At present the arrangements are only of a provisional character as they must apparently obtain the sanction of the Insurance Commissioners before they can be carried into effect. We trust, however, that there will not be any difficulty in obtaining the approval of the Commissioners, for the scheme recognises the essential value of efficient home-nursing and is calculated to remove the baneful influence of malingering.

If approved, the experiment will be watched with interest, and should it be successful there is every reason to suppose that its scope would be extended far beyond the boundaries of the County of Kent.

**NOTE.—Questions and Correspondence on all doubtful points are invited, and should be addressed to the Editor, The Hospital, 28, 29 Southampton Street, Strand, London, W.C., and marked "Insurance."**

